# Educational attainment and self-reported environmental exposures of pregnant women living in Nairobi, Kenya

**DOI:** 10.1371/journal.pgph.0005453

**Published:** 2025-11-18

**Authors:** Christopher Zuidema, Priscillah Wanini Edemba, Anne M. Riederer, Vincent K. Kipter, Allison R. Sherris, Lewis Olweywe, Judith Adhiambo, Brendah Isavwa, Erica A. Wetzler, Barbra A. Richardson, John Kinuthia, Michael J. Gatari, Elizabeth Maleche-Obimbo, Edmund Seto, Catherine J. Karr, Sarah Benki-Nugent, Faridah H. Were

**Affiliations:** 1 Department of Environmental and Occupational Health Sciences, University of Washington, Seattle, Washington, United States of America; 2 Department of Medicine, Division of Public Health, University of Vermont, Burlington, Vermont, United States of America; 3 Kenyatta National Hospital, Nairobi, Kenya; 4 Department of Chemistry, Faculty of Science and Technology, University of Nairobi, Nairobi, Kenya; 5 Department of Paediatrics and Child Health, University of Nairobi, Nairobi, Kenya; 6 Department of Global Health, University of Washington, Seattle, Washington, United States of America; 7 Department of Biostatistics, University of Washington, Seattle, Washington, United States of America; 8 Institute of Nuclear Sciences, University of Nairobi, Nairobi, Kenya; 9 Department of Pediatrics, University of Washington, Seattle, Washington, United States of America; 10 Department of Medicine, Division of Allergy and Infectious Diseases, University of Washington, Seattle, Washington, United States of America; Sustainable Futures Collaborative, INDIA

## Abstract

Environmental exposures experienced by pregnant women living in cities of low- and middle-income countries are poorly described. We collected information on housing characteristics, household and ambient air pollution, and work-related exposures through questionnaires in a cohort of 400 pregnant women in Nairobi, Kenya and examined exposures in relation to educational attainment, a proxy for socioeconomic status. Participants were median 26 years of age, mostly married (85%) and self-described homemakers (54%), with at least some secondary school education (74%). Housing generally consisted of one room (58%) with one window (57%), access to electricity (94%), and no piped water (88%). Participants commonly reported living near sewers (55%) and the Dandora dumpsite (34%). Liquified petroleum gas (LPG) was the primary domestic cooking fuel, followed by kerosene, with 65% and 32%, respectively, reporting use most days to daily. Most participants reported exposure to outdoor cooking smoke (73%) and vehicle exhaust (66%) most days to daily. Employed participants (N = 151) reported work-related exposure to vehicle exhaust (68%), cigarettes (37%), marijuana (33%), dusty/unpaved roads (32%), welding (30%), and the dumpsite (30%) most days to daily. Exposure to rubbish burning was reported by 4.5% of participants at home, by 24% outdoors, and by 25% related to work most days to daily. Relative to women with primary school education or lower, women with at least secondary school education were more likely to use LPG (p < 0.001) and less likely to use kerosene (p < 0.001). This study highlights a high prevalence of pregnant women in an urban African context experience multiple sources of combustion related air pollution sources. Exposure to cooking emissions, vehicle exhaust, and rubbish burning were frequent. Kerosene, the second most common and a dirty domestic fuel, was used more by women with lower educational attainment. These data may inform future studies of prenatal and early childhood environmental exposures and interventions.

## Introduction

Environmental conditions experienced during pregnancy, a critical period of susceptibility, are important drivers of birth outcomes and infant/child health outcomes [1–3]. Cities in low- and middle-income countries (LMICs) are growing rapidly, putting pressure on resources, and compounding environmental exposures and associated health risks [4,5]. Key environmental exposures in cities are encountered in the home, ambient environment, or through work and include poor quality housing, inadequate access to sanitation and clean water, household fuel combustion, environmental tobacco smoke, transportation and roadway emissions, unregulated industrial/commercial activities, hazardous waste and rubbish dumping and burning, and other sources of ambient air pollution (AAP) [6–12].

Important air pollutants in cities include combustion related particulate matter (PM), carbon monoxide (CO), nitrogen dioxide (NO_2_), and black carbon (BC) [8,13]. A broad range of adverse health effects are related to these pollutants including respiratory, pulmonary, and cardiovascular disease, infection, cancer, and death [12,14–16]. For pregnant women in particular, exposure to PM is associated with low birth weight, preterm birth, stillbirth and small for gestational age [17–19]. Beyond birth outcomes, prenatal air pollution exposure is associated with adverse respiratory, immune, cardiometabolic, and neurodevelopmental effects in children [20,21].

In sub-Saharan Africa there has been extensive study of solid fuel cookstove emissions and household air pollution (HAP) in rural settings, which disproportionately impacts women [15,22,23]. However, the impact of ambient and urban air pollution is increasing [24,25] and less research has focused on women’s environmental health experiences in dense urban settings. While urban residents may be aware of hazardous environmental exposures, they may also underestimate the magnitude of the problem, they may have different perceptions based on their proximity to pollution sources, and they may not connect air pollutant exposure to health effects [12]. Furthermore, lower socioeconomic status (SES) has been linked with a range of increased environmental risk factors [26,27]. In North America, for example, lower SES is associated with higher AAP exposure [28], and although research in LMICs has shown similar trends [29,30], the number of studies, particularly in Africa, is limited [5,25,28].

Examining the perceived environmental and occupational health exposures of pregnant women and their relation to socioeconomic factors, such as educational attainment, is essential to understanding the risks they and their families face while designing suitable intervention and control strategies. In addition, characterizing these conditions can motivate environmental epidemiological analyses that are rare in sub-Saharan Africa [31] and inform prioritization by health and environmental policy-makers. To date, research on environmental factors and the unique vulnerability of pregnant women, whose exposures have important consequences on fetal development, in urban sub-Saharan settings is lagging. The first goal of this study was to describe self-reported environmental and occupational health exposures among Nairobi women enrolled in a pregnancy cohort established in 2022. We focus on housing characteristics; HAP, highlighting household fuels; sources of AAP; and work-related exposures. Our second goal was to explore the relationship between these environmental exposures and education, a well-known proxy for SES in health and epidemiologic studies [32–34], which may also influence choices and behaviors affecting environmental exposures. We hypothesized that higher educational attainment would be associated with more frequent use of cleaner household fuels, higher quality housing characteristics, and less frequent reporting of adverse environmental exposures.

## Methods

### Ethics statement

All study procedures were approved by the University of Washington Institutional Review Board and the Kenyatta National Hospital - University of Nairobi Ethics and Research Committee. Written informed consent was obtained from eligible women prior to enrollment. Additional information regarding the ethical, cultural, and scientific considerations specific to inclusivity in global research is included in the Supporting Information (S1 Checklist).

### Data description

The study participants are part of the Air Pollution Exposures in Early Life and Brain Development in Children (ABC) study. ABC is an ongoing prospective cohort study of 400 mother-child pairs investigating prenatal exposure to combustion related air pollutants (including polycyclic aromatic hydrocarbons, fine particulate matter (PM_2.5_), ultrafine particulate, BC, and CO) and child neurodevelopment through 36 months of the child’s age [35]. Participants were recruited and enrolled from the Dandora II Health Centre, a facility that serves a patient population of the Dandora neighborhood in Nairobi, Kenya from January 10 to November 29, 2022.

We collected data with a structured enrollment questionnaire administered by research team members. The questionnaire included sections on: (i) maternal demographics (e.g., age, education, employment, marital status), (ii) housing characteristics, including construction materials, building systems, and potential hazards located in or near the residence (e.g., ventilation, mold, pests, nearby industrial sources), (iii) household fuels and other sources of HAP (e.g., incense, smoking), (iv) sources of ambient (outdoor) air pollution, generally located within 1 km of the residence (e.g., vehicle exhaust, dumpsite pollution) and within 20 m for the participant’s outdoor cooking smoke and their neighbors’ cooking smoke, and (v) work-related or occupational exposures among employed participants. The full enrollment questionnaire is provided in the Supporting Information (S1 File).

### Data analysis

We used educational attainment as a measure of SES because information about education is relatively simple to collect in questionnaires, is relevant regardless of age or employment status, is comparable across studies, and avoids social stigma associated with other measures, such as income or rent information [32,33]. We grouped participants into two educational categories: those with lower educational attainment whose highest education level was less than secondary school (i.e., never went to school, or highest education level was preprimary or primary school) and those with higher educational attainment whose highest education level was at least secondary school (i.e., highest education level was secondary school, vocational school, or college/university).

Many of the questions were framed as frequency factors, where participants reported sources of exposure occurring not at all, rarely, some days, most days, or daily. We consolidated these frequency responses into three categories: not at all to rarely, some days, and most days to daily. We tabulated the demographic, housing, HAP, AAP, and work-related responses for all participants and for educational attainment groups separately and conducted Fisher’s exact test (for counts <5) or Pearson’s chi-squared tests (for counts ≥5) for hypothesis testing. Our criterion for statistical significance was p ≤ 0.05. We also prepared frequency plots to summarize the data. Data analysis was carried out in R version 4.4.1.

## Results

### Maternal demographic characteristics

The median (25^th^, 75^th^ percentile) age among enrolled participants (N = 400) was 26 (23, 31) years (Table 1). Most participants lived in the Dandora neighborhood. Median parity was 1 (0, 2). One hundred four (26%) participants reported their highest level of education was less than secondary school (i.e., they had lower educational attainment), while 58% reported their highest level of education was secondary school and 16% vocational or college/university (i.e., N = 296 (74%) had higher educational attainment). Most women reported they were a “homemaker (housewife)” (54%) or were not employed (8%). Among participants that reported employment (N = 151), the most common type was small business (45%), followed by daily wage (31%) and monthly salary (24%). Most women reported being married (85%), followed by never married/single (11%) and their main financial supporter was their husband, ex-husband, boyfriend, or child’s father (82%). Several women reported themselves as the main financial supporter (12%).

**Table 1:** Demographic characteristics at study enrollment (N = 400).

Characteristic	N (%) or median (25^th^, 75^th^ percentile)
Age	26.0 (23, 31)
Trimester enrolled	
First trimester	14 (3.5%)
Second trimester	182 (46%)
Third trimester	204 (51%)
Parity	
0	104 (26%)
1	132 (33%)
2	93 (23%)
≥ 3	71 (18%)
Employment status	
Homemaker (housewife)	216 (54%)
Small business	68 (17%)
Daily wage	47 (12%)
Monthly salary	36 (9.0%)
Not employed	33 (8.3%)
Marital status	
Married	339 (85%)
Never married/ single	45 (11%)
Divorced/separated, widowed	16 (4.0%)
Main financial supporter	
Husband, ex-husband, boyfriend, or child’s father	328 (82%)
Self	47 (12%)
Parent	19 (4.8%)
Other relative	6 (1.5%)
Educational attainment	
< Secondary	104 (26%)
≥ Secondary	296 (74%)

### Housing characteristics – construction materials, building systems, and proximity to potential hazards

Eighty-three percent of participants rented their house and reported a median monthly rent of KES 3,500 (2,500, 5,000) (approximately US $30); 6.3% owned their house (Table 2). The median duration of stay in their current residence was 14 (5, 36) months, while 5.5% reported stays of ten years or more. The median number of people per room was 2.0 (1.5, 3.0). Most houses had one room (58%), one window (57%), and were constructed with concrete floors (75%), stone masonry walls (94%), and mabati/iron sheet roof (85%). Other materials reported included stone or polyvinyl chloride (PVC) tile floors, metal walls, and reinforced concrete slab roof. Nearly all participants reported their residence had access to electricity (94%). While many participants reported using flush toilets (97%), few had access to piped water indoors (12%) and 96% stored domestic water in plastic containers. In the kitchen area, most participants did not report chimneys for cooking stoves (N = 398), though most reported kitchen doors, windows, and wall vents (98%) to ventilate and exhaust cooking emissions. Pests such as cockroaches or rats were commonly reported (71%), though chipping or peeling paint was less common (26%). Participants also reported living “near” potential environmental hazards (Table 2). Living near sewers (55%) and the Dandora dumpsite (34%) were the most commonly reported proximal hazards, followed by automotive garages (20%), polluted rivers (19%), electronic waste recycling activity (17%), factories (9.3%), and battery recycling (7.3%).

**Table 2:** Self-reported social, physical, and proximal housing characteristics by educational attainment.

Housing characteristic N (%) or median (25^th^, 75^th^ percentile)	Total N = 400	< Secondary Education N = 104	≥ Secondary Education N = 296	p-value
Social				
Rents house	330 (83%)	87 (84%)	243 (82%)	0.7
Household rent (KES)	3,500 (2,500, 5,000)	3,000 (2,500, 4,000)	3,500 (3,000, 5,000)	<0.001
Stay duration (months)	14 (5, 36)	24 (8, 51)	12 (5, 36)	0.002
People per room	2.0 (1.5, 3.0)	3.0 (2.0, 4.0)	2.0 (1.5, 3.0)	<0.001
Physical				
Rooms in house				0.3
1	231 (58%)	64 (62%)	167 (56%)	
2	120 (30%)	32 (31%)	88 (30%)	
≥ 3	49 (12%)	8 (7.7%)	41 (14%)	
Windows in house				0.02
0	10 (2.5%)	5 (4.8%)	5 (1.7%)	
1	226 (57%)	66 (63%)	160 (54%)	
≥ 2	164 (41%)	33 (32%)	131 (44%)	
Kitchen ventilation (chimney, door, windows, vents)	393 (98%)	102 (98%)	291 (98%)	>0.9
Electricity access	376 (94%)	93 (89%)	283 (96%)	0.02
Piped water, outside house	349 (87%)	96 (92%)	253 (85%)	0.07
Plastic water storage container	384 (96%)	103 (99%)	281 (95%)	0.08
Flush toilet	388 (97%)	99 (95%)	289 (98%)	0.3
Concrete floor	299 (75%)	79 (76%)	220 (74%)	0.7
Stone walls	375 (94%)	94 (90%)	281 (95%)	0.10
Mabati/iron sheet roof	338 (85%)	91 (88%)	247 (83%)	0.3
Mold, visible or odor	92 (23%)	24 (23%)	68 (23%)	>0.9
Pests (e.g., cockroaches, rats)	284 (71%)	72 (69%)	212 (72%)	0.6
Chipping or peeling paint, floors/walls	105 (26%)	26 (25%)	79 (27%)	0.7
Proximal				
Near sewers	219 (55%)	56 (54%)	163 (55%)	0.8
Near dumpsite	137 (34%)	37 (36%)	100 (34%)	0.7
Near garage	80 (20%)	17 (16%)	63(21%)	0.3
Near polluted river	77 (19%)	24 (23%)	53 (18%)	0.2
Near electronic waste recycling	68 (17%)	20 (19%)	48 (16%)	0.5
Near factory	37 (9.3%)	12 (12%)	25 (8.4%)	0.3
Near battery recycling	29 (7.3%)	9 (8.7%)	20 (6.8%)	0.5
Near none of these	85 (21%)	16 (15%)	69 (23%)	0.09

Participants with higher educational attainment paid higher rent (p < 0.001), reported significantly fewer people per room (p < 0.001), a greater number of windows (p = 0.02), greater electricity access (p = 0.02), and a shorter duration staying in their residence (p = 0.002) compared to participants with lower educational attainment (Table 2). While not statistically significant, participants with lower educational attainment were more likely to report storing water in plastic jugs and accessing piped water from source outside of their home. Participants with higher educational attainment also reported living near “none of these” potential hazards more frequently than those with lower educational attainment, though this difference was not statistically significant (p = 0.09).

### Household fuels and other household air pollution

**Fig 1:**
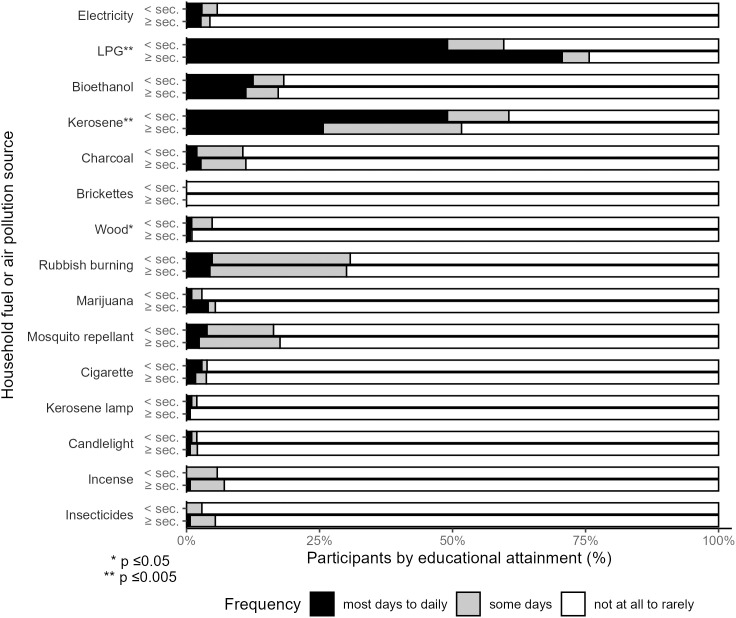
Self-reported use of household fuels and sources of household air pollution (N = 400).

Most participants lived in one-room houses, putting cooking in close proximity to other domestic activities. There was low reported use of electricity for cooking, with 11 participants (2.8%) reporting use most days to daily (Fig 1 and S1 Table in S2 File). Liquified petroleum gas (LPG) was the most frequently used household fuel, with 65% of participants reporting use most days to daily. Bioethanol fuel was used by 12% of participants most days to daily. Many participants also reported using higher emission fuels like kerosene most days to daily (32%) or some days (22%). There was little use of solid fuels in this cohort, with 10 (2.5%) participants reporting using charcoal and 3 reporting using wood most days to daily. Forty-one percent reported using dirtier fuels (wood, kerosene, charcoal, or brickettes) rarely to not at all. Some participants also reported using more than one fuel; for example, among the 260 participants who reported using LPG most days to daily, 10% also reported also using kerosene most days to daily. Household fuel usage differed by educational attainment; those with higher educational attainment were more likely to use LPG (p < 0.001) and less likely to use kerosene (p < 0.001) than participants with lower educational attainment (S1 Table in S2 File).

Generally, participants reported few other non-cooking sources of HAP (Fig 1 and S1 Table in S2 File). The most common HAP source was indoor rubbish burning, with 4.5% reporting this activity most days to daily and 26% reporting it some days, while 70% of participants reported it rarely to not at all. Mosquito repellant was used rarely to not at all by most participants (83%), as were insecticides (95%) and incense (93%). More than 95% reported marijuana and cigarette smoke, burning candles, and kerosene lamp use rarely to not at all, though 52 participants (13%) reported at least one person smoked cigarettes or marijuana in the home. Among participants primarily using LPG, 50% reported no other HAP exposures. There were no differences in HAP exposures by educational attainment.

### Ambient (outdoor) air pollution

**Fig 2:**
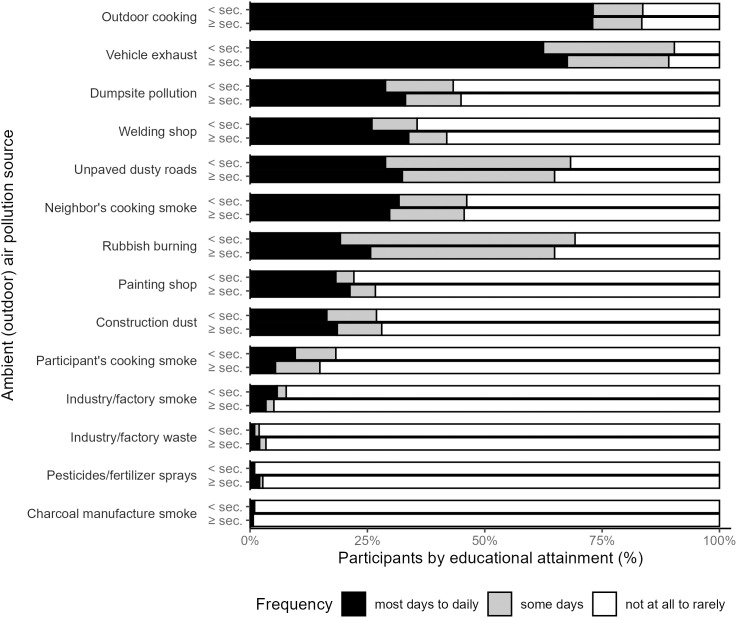
Self-reported sources of ambient (outdoor) air pollution exposures reported within 1 km of participant residence (note: neighbor’s and participant’s cooking smoke within 20 m) (N = 400).

Participants reported a variety of AAP sources (Fig 2 and S2 Table in S2 File). Within 20 m of their residence, most participants reported they do not experience smoke from their own outdoor cooking (84% rarely to not at all), but nearly one-third did report exposure to smoke from their neighbors’ cooking (30% most days to daily). In addition, within 1 km of their residence, 73% percent of participants reported being exposed to outdoor cooking smoke most days to daily. Other frequently reported ambient exposures within 1 km of their residence were vehicle exhaust (66%), dumpsite pollution (32%), welding shop activity (32%), and unpaved/dusty roads (32%) most days to daily. There were no differences in reported ambient air pollution exposure frequency by educational attainment (S2 Table in S2 File).

### Work-related and occupational exposures

**Fig 3:**
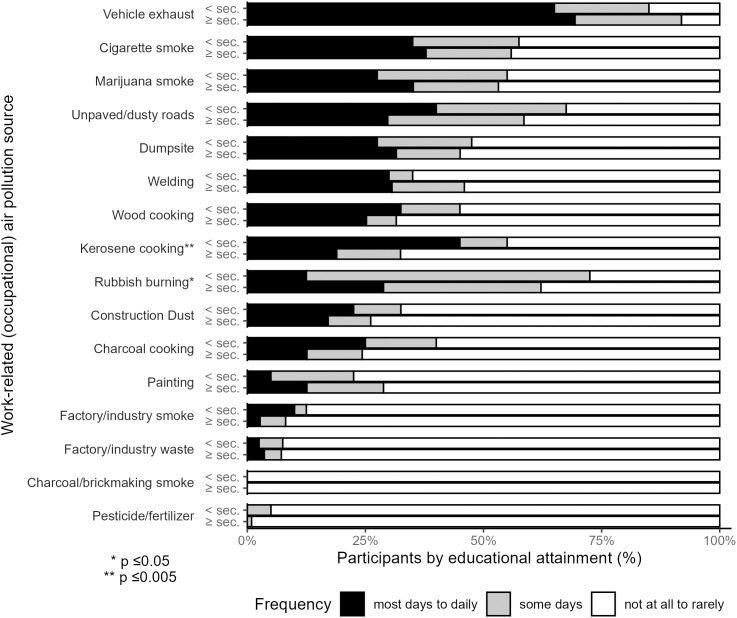
Self-reported sources of work-related (occupational) air pollution exposure (N = 151).

Employed participants (N = 151) reported work exposures related to vehicles and roadways, secondhand smoke, cooking, and waste management (Fig 3 and S3 Table in S2 File). Sixty-eight percent of participants reported exposure to vehicle exhaust and 32% to unpaved/dusty roads most days to daily. Exposure most days to daily to cigarettes (37%) and marijuana (33%) was commonly reported. Work-related exposure to cooking emissions, including wood (27%), kerosene (26%), and charcoal (16%) were also frequently reported most days to daily. Thirty percent of employed participants reported work-related exposure to the dumpsite most days to daily; for rubbish burning, 25% reported exposure most days to daily, while 40% reported exposure some days. Welding shop activity was also a frequent work-related exposure, with 30% reporting exposure most days to daily. Notably, few participants reported exposures related to charcoal brickette manufacture, pesticide/fertilizer, or industry/factory smoke or waste (≤ 5% most days to daily).

Participants with lower educational attainment were more likely to report work-related kerosene cooking exposures (p = 0.005). Reported work-related exposure to rubbish burning also differed by educational attainment (p = 0.01); participants with lower educational attainment were more likely to report exposure some days, but less likely to report it not at all to rarely or most days to daily (S3 Table in S2 File). Other work-related exposures did not differ by educational attainment.

## Discussion

Our results show that pregnant women living in Nairobi often face a variety of environmental exposures, regardless of educational attainment. In addition, women with lower educational attainment had greater exposure to certain pollutants. Overall, cooking emissions, vehicle exhaust, road dust, the Dandora dumpsite, secondhand smoke, pests, rubbish burning, and proximity to sewers were exposures most frequently reported by participants in residential, ambient, and work settings.

As expected, participants with higher educational attainment were more likely to earn a monthly salary and pay more rent, supporting the use of educational groups as a proxy for SES. Higher educational attainment may be related to some of the choices and behaviors that influence environmental exposures. For example, those with higher educational attainment had stays half as long as those with lower educational attainment, potentially reflecting a means to change their residence motivated by the pregnancy. Participants with higher educational attainment were more likely to report using LPG and less likely to report using kerosene in their own house. Similarly, participants with higher educational attainment were less likely to report work-related kerosene cooking exposures. Work-related exposure to rubbish burning also differed by educational attainment. However, higher educational attainment was less closely linked with exposures related to residential proximity (e.g., sewers and dumpsite), HAP (e.g., rubbish burning), AAP (e.g., cooking smoke and vehicle exhaust), and work (e.g., charcoal and wood cooking), as might be expected.

Studies in other African geographies offer important context for our observations. In a review of environmental health research on the continent, ambient air pollution, indoor air pollution, heavy metals, pesticides, and dietary mold were among the exposures most studied [36] and mirrored the exposures most often reported by participants in our study. In a study of fetal growth among healthy pregnant women at low risk of adverse pregnancy outcomes and environmental exposures, indicators of environmental health among Nairobi participants were somewhat better than our observations a decade later [37]. For example, housing was less dense (median 0.5 people per room versus 2 people per room), < 5% reporting mold/mildew odors (compared to 23% in this study), 18% reported peeling paint (compared to 26% observed in the current study), just over half reported pests such as mice rats and cockroaches (compared to 71% in this study), and 88.4% had gas and 9.4% had electric stoves [37], compared to the 65% using LPG and 2.8% using electricity most days to daily in our study.

Beyond Kenya, a systematic review in Ethiopia examined exposures during pregnancy and birth outcomes and reported that biomass fuels, environmental tobacco smoke, and pesticides were among those most commonly experienced and associated adverse birth outcomes [38]. Studies in Durban and Bloemfontein, South Africa examining birth outcomes, reported much higher use of electricity as a cooking energy source (> 90%) than we observed, though nearly all participants also reported cooking indoors exclusively [39,40]. However, smoking tobacco indoors was more prevalent in one of these studies compared to our results, where 49% of households permitted smoking indoors and 76% reported exposure to environmental tobacco smoke indoors [39], compared to our results where 95% reported exposure rarely to not at all and 13% reported a smoker in the home. Also more prevalent, was proximity to industrial sources (15%) [39]. Further study of the awareness, resources, and agency to reduce environmental exposures among pregnant women in sub-Saharan Africa is warranted.

Housing characteristics influence indoor air quality and HAP, as well as water and sanitation behaviors, which can contribute to poor environmental health conditions [41,42]. Nearly all participants had access to improved water sources (piped water) and sanitation (flush toilets rather than latrines) at or near their house – positive features for environmental health. However, one room houses were common and may not provide sufficient living space and increase risk of disease transmission and increased concentrations of pollutants. Furthermore, while many houses had windows and ventilation, emission-generating activities like cooking still occur near all other aspects of domestic life. Pests (such as cockroaches and rats) and mold were reported by 71% and 23% of participants, respectively, contributing to poor indoor air quality conditions. Twenty-six percent of participants reported chipping or peeling paint, which could pose a lead hazard despite the 2018 ban on lead in paint [43]. Half of participants also reported living in their residence less than fourteen months, potentially indicating precarity of tenure and housing quality.

Household fuel use is among the most important factors influencing HAP. In contrast to rural Kenyans, where over 90% rely mostly on wood [44,45], few participants reported wood use daily or most days (<1%). Despite this lower use of solid fuels, common urban fuels may also produce harmful emissions. One 2012–2014 study in Nairobi reported kerosene was the predominant fuel among 69.7% of residents in the informal settlements of Korogocho and Viwandani [46]. Ten years later in the current study, 32% reported kerosene use most days to daily, with higher usage (49%) among participants with lower educational attainment. Instead of solid fuels or kerosene, LPG was used by 65% of participants most days or daily, which is comparable to the 60% reported in the 2022 Kenya Demographic and Health Survey [47]. Such LPG use reflects the broader trend of LPG becoming a more common fuel in LMICs as international campaigns and governments promote cleaner fuels [48,49]. Furthermore, urbanization, socioeconomic status, educational attainment, and income have been associated with greater adoption of LPG [50,51], which may partly explain the high use of LPG among participants.

Cleaner stoves are not without risk, however; LPG users, particularly in low ventilation settings, may still be exposed to PM concentrations that exceed World Health Organization guidelines [52], as well as other contaminants including NO_2_ [53], benzene [54], and a range of other hazardous air pollutants [55] which pose risks to human health. Additionally, users of cleaner cooking fuels may also use alternative fuels, such as kerosene, charcoal, or wood in fuel stacking [50,56]. In this study, 26 participants reported using both LPG and kerosene most days to daily. Kerosene cooking fuel use is associated with increased respiratory morbidity, adverse blood pressure effects, and mortality [57]. Beyond HAP, ambient air quality guidelines may not be achieved due to the practice of fuel stacking [7].

Despite the high proportion of women that reported using cleaner fuels in their own house, 73% reported being frequently exposed to outdoor cooking pollution, potentially from neighbors or businesses. Participants also frequently reported AAP sources including vehicle exhaust (66%), welding shop activity (32%), unpaved/dusty roads (32%), the Dandora dumpsite (32%), and rubbish burning (24%) within 1 km of their residence. Similar themes emerged from other recent reports of perceived air pollution, where rubbish burning, cooking fuels, dump sites, and industry were top concerns [58,59]. In contrast, in this study industry was one of the least reported sources of exposure (4%). In a 2013 study in Nairobi, researchers observed low awareness of air pollution, with 35–55% having never heard information about air pollution exposure, despite the common practice of waste burning from a nearby dumpsite [58]. Our results possibly indicate a growing awareness of environmental exposures over time.

Main work-related or occupational exposures reported by participants were vehicle exhaust and unpaved/dusty roads; this experience is supported by prior research in which women working alongside roads and potentially walking to work were exposed to PM_2.5_ concentrations of 62.7 ± 20.5 µg/m^3^ from resuspended road dust and refuse burning in Nairobi [60]. Another study linked work as a street vendor with a reduction in birth weight [61]. In the present study, a common work-related exposure was smoke from tobacco and marijuana, with 52% of participants reporting exposure at least some days, despite there being few active smokers in this study (1.8%), and smoke from marijuana and cigarettes in the house not being frequently identified (3.8% reported exposure at least some days). Exposure to cigarette smoke only (56%) was higher in our results than other studies where 12–19% [58] and 3.2% [46] of Nairobi women reported exposure to cigarette smoke in the workplace. In the same study, 14.7% of participants reported one or more members of their household smoked, with 6.7% reporting smoking inside the house [46]. Our results highlight that despite being a well-recognized prenatal exposure of concern that should be eliminated, exposure to secondhand smoke is still prevalent in Nairobi among pregnant women, especially in work settings.

Exposure to rubbish burning is an exposure of particular concern, occurring within households, outdoors, and in work contexts [62,63]. Encouragingly, 70% of participants reported burning rubbish at home rarely to not at all. For the 26% of participants that reported this practice some days and 4.5% reporting it most days to daily, there is an opportunity for education to reduce this potentially hazardous exposure. A higher percentage of participants reported exposure to rubbish burning in the ambient environment and workplace (~40% some days and ~25% most days to daily). Reducing these exposures could be more challenging, potentially requiring community action, and is compounded by the proximity and operations of the Dandora dumpsite.

Seventy-four percent of participants reported their highest level of education was secondary school, compared with 50% of women in the 2022 Kenyan Demographic and Health Survey [47]. This difference, combined with the potential for participants’ spouses to be similarly educated [64,65], suggests relatively higher SES among women in this cohort and potentially limited generalizability of our results. Similarly, participants generally lived within a specific neighborhood and their experiences may not be shared by pregnant women in other parts of Nairobi or other cities in sub-Saharan Africa. Another limitation of this study was that women that opted to participate in the study may differ from those who did not, possibly causing selection bias. Finally, data were based on participant self-report and recall, which could be biased if participants were less aware of certain pollution sources, provided answers they felt were socially appropriate, or inaccurately recalled their practices and behaviors. Despite these potential limitations, our results provide useful insights into environmental health conditions experienced by the pregnant women in this study.

Our findings - that pregnant women in Nairobi face a variety of environmental exposures and that lower educational attainment is associated with greater exposure to certain pollutants - build upon the limited understanding of residential, ambient, and work-related environmental risk factors for this population. Furthermore, improving understanding of social and economic factors that are linked to housing conditions, household fuel use, HAP, AAP, and work-related exposures is critical for focusing interventions and control measures that are more likely to have sustained and effective reductions for individuals at highest risk. However, despite its importance, government entities and budgets to address environmental hazards in pregnancy, particularly for HAP [66], are lacking. Continued efforts throughout sub-Saharan Africa are needed to identify environmental health priorities for pregnant women for well-informed policy.

## Supporting information

S1 File Data collection questionnaire. (PDF)

S2 File S1 Table. Self-reported household fuels and sources of household air pollution exposure. **S2 Table**. Self-reported sources of ambient (outdoor) air pollution exposure. **S3 Table**. Self-reported sources of work-related air pollution exposure.
(PDF)

S1 Checklist Inclusivity in global research. (DOCX)

S1 Data Anonymized dataset. (CSV)
