# Supplementary material for: Educational attainment and self-reported environmental exposures of pregnant women living in Nairobi, Kenya
Source: PLOS Glob Public Health. 2025 Nov 18;5(11):e0005453. doi: 10.1371/journal.pgph.0005453 (PMC12626295; doi:10.1371/journal.pgph.0005453)
Supplement: S1 File — (PDF) [file pgph.0005453.s001.pdf]

Educational attainment and self-reported environmental exposures of pregnant women living in Nairobi, Kenya

Christopher Zuidema, Priscillah Wanini Edemba, Anne M. Riederer, Vincent K. Kipter, Allison R. Sherris, Lewis Olweywe, Judith Adhiambo, Brendah Isavwa, Erica A. Wetzler, Barbra A. Richardson, John Kinuthia, Michael J. Gatari, Elizabeth Maleche-Obimbo, Edmund Seto, Catherine J. Karr, Sarah Benki-Nugent, Faridah H. Were

Tables

S1 Table. Self-reported household fuels and sources of household air pollution exposure.

Fuel or household air pollutant	Total<br>N = 400	< Secondary Education<br>N = 104	≥ Secondary Education<br>N = 296	p-value
Electricity				0.7
not at all to rarely	381 (95%)	98 (94%)	283 (96%)	
some days	8 (2.0%)	3 (2.9%)	5 (1.7%)	
most days to daily	11 (2.8%)	3 (2.9%)	8 (2.7)	
LPG				<0.001
not at all to rarely	114 (29%)	42 (40%)	72 (24%)	
some days	26 (6.5%)	11 (11%)	15 (5.1%)	
most days to daily	260 (65%)	51 (49%)	209 (71%)	
Bioethanol				>0.9
not at all to rarely	330 (83%)	85 (82%)	245 (83%)	
some days	24 (6.0%)	6 (5.8%)	18 (6.1%)	
most days to daily	46 (12%)	13 (13%)	33 (11%)	
Kerosene				<0.001
not at all to rarely	184 (46%)	41 (39%)	143 (48%)	
some days	89 (22%)	12 (12%)	77 (26%)	
most days to daily	127 (32%)	51 (49%)	76 (26%)	
Charcoal				>0.9
not at all to rarely	356 (89%)	93 (89%)	263 (89%)	
some days	34 (8.5%)	9 (8.7%)	25 (8.4%)	
most days to daily	10 (2.5%)	2 (1.9%)	8 (2.7%)	
Brickettes				>0.9
not at all to rarely	400 (100%)	104 (100%)	296 (100%)	
some days	0 (0%)	0 (0%)	0 (0%)	
most days to daily	0 (0%)	0 (0%)	0 (0%)	
Wood				0.03
not at all to rarely	392 (98%)	99 (95%)	293 (99%)	
some days	5 (1.3%)	4 (3.8%)	1 (0.3%)	
most days to daily	3 (0.8%)	1 (1.0%)	2 (0.7%)	
Rubbish burning				>0.9
not at all to rarely	279 (70%)	72 (69%)	207 (70%)	
some days	103 (26%)	27 (26%)	76 (26%)	
most days to daily	18 (4.5%)	5 (4.8%)	13 (4.4%)	
Marijuana				0.2
not at all to rarely	381 (95%)	101 (97%)	280 (95%)	
some days	6 (1.5%)	2 (1.9%)	4 (1.4%)	
most days to daily	13 (3.3%)	1 (1.0%)	12 (4.1%)	
Mosquito repellant				0.6
not at all to rarely	331 (83%)	87 (84%)	244 (82%)	
some days	58 (15%)	13 (13%)	45 (15%)	
most days to daily	11 (2.8%)	4 (3.8%)	7 (2.4%)	
Cigarette				0.6
not at all to rarely	385 (96%)	100 (96%)	285 (96%)	
some days	7 (1.8%)	1 (1.0%)	6 (2.0%)	
most days to daily	8 (2.0%)	3 (2.9%)	5 (1.7%)	
Kerosene lamp				0.4
not at all to rarely	396 (99%)	102 (98%)	294 (99%)	

some days	1 (0.3%)	1 (1.0%)	0 (0%)	
most days to daily	3 (0.8%)	1 (1.0%)	2 (0.7%)	
Candlelight				>0.9
not at all to rarely	392 (98%)	102 (98%)	290 (98%)	
some days	5 (1.3%)	1 (1.0%)	4 (1.4%)	
most days to daily	3 (0.8%)	1 (1.0%)	2 (0.7%)	
Incense				>0.9
not at all to rarely	373 (93%)	98 (94%)	275 (93%)	
some days	25 (6.3%)	6 (5.8%)	19 (6.4%)	
most days to daily	2 (0.5%)	0 (0%)	2 (0.7%)	
Insecticides				0.8
not at all to rarely	381 (95%)	101 (97%)	280 (95%)	
some days	17 (4.3%)	3 (2.9%)	14 (4.7%)	
most days to daily	2 (0.5%)	0 (0%)	2 (0.7%)	

S2 Table. Self-reported sources of ambient (outdoor) air pollution exposure.

Ambient air pollutant	Total<br>N = 400	< Secondary Education<br>N = 104	≥ Secondary Education<br>N = 296	p-value
Outdoor cooking				>0.9
not at all to rarely	66 (17%)	17 (16%)	49 (17%)	
some days	42 (11%)	11 (11%)	31 (10%)	
most days to daily	292 (73%)	76 (73%)	216 (73%)	
Vehicle exhaust				0.4
not at all to rarely	42 (11%)	10 (9.6%)	32 (11%)	
some days	93 (23%)	29 (28%)	64 (22%)	
most days to daily	265 (66%)	65 (63%)	200 (68%)	
Dumpsite pollution				0.6
not at all to rarely	222 (56%)	59 (57%)	163 (55%)	
some days	50 (13%)	15 (14%)	35 (12%)	
most days to daily	128 (32%)	30 (29%)	98 (33%)	
Welding shop				0.3
not at all to rarely	239 (60%)	67 (64%)	172 (58%)	
some days	34 (8.5%)	10 (9.6%)	24 (8.1%)	
most days to daily	127 (32%)	27 (26%)	100 (34%)	
Unpaved dusty roads				0.4
not at all to rarely	137 (34%)	33 (32%)	104 (35%)	
some days	137 (34%)	41 (39%)	96 (32%)	
most days to daily	126 (32%)	30 (29%)	96 (32%)	
Neighbor's cooking smoke				0.9
not at all to rarely	217 (54%)	56 (54%)	161 (54%)	
some days	62 (16%)	15 (14%)	47 (16%)	
most days to daily	121 (30%)	33 (32%)	88 (30%)	
Rubbish burning				0.14
not at all to rarely	136 (34%)	32 (31%)	104 (35%)	
some days	168 (42%)	52 (50%)	116 (39%)	
most days to daily	96 (24%)	20 (19%)	76 (26%)	
Painting shop				0.6
not at all to rarely	298 (75%)	81 (78%)	217 (73%)	
some days	20 (5.0%)	4 (3.8%)	16 (5.4%)	
most days to daily	82 (21%)	19 (18%)	63 (21%)	
Construction dust				0.8
not at all to rarely	289 (72%)	76 (73%)	213 (72%)	
some days	39 (9.8%)	11 (11%)	28 (9.5%)	
most days to daily	72 (18%)	17 (16%)	55 (19%)	
Participant's cooking smoke				0.3
not at all to rarely	337 (84%)	85 (82%)	252 (85%)	
some days	37 (9.3%)	9 (8.7%)	28 (9.5%)	
most days to daily	26 (6.5%)	10 (9.6%)	16 (5.4%)	
Industry/factory smoke				0.6
not at all to rarely	377 (94%)	96 (92%)	281 (95%)	
some days	7 (1.8%)	2 (1.9%)	5 (1.7%)	
most days to daily	16 (4.0%)	6 (5.8%)	10 (3.4%)	
Industry/factory waste				0.9
not at all to rarely	388 (97%)	102 (98%)	286 (97%)	
some days	5 (1.3%)	1 (1.0%)	4 (1.4%)	
most days to daily	7 (1.8%)	1 (1.0%)	6 (2.0%)	
Pesticides/fertilizer sprays				0.8
not at all to rarely	391 (98%)	103 (99%)	288 (97%)	
some days	2 (0.5%)	0 (0%)	2 (0.7%)	
most days to daily	7 (1.8%)	1 (1.0%)	6 (2.0%)	
Charcoal manufacture smoke				0.6
not at all to rarely	397 (99%)	103 (99%)	294 (99%)	
some days	1 (0.3%)	0 (0%)	1 (0.3%)	
most days to daily	2 (0.5%)	1 (1.0%)	1 (0.3%)	

S3 Table. Self-reported sources of work-related air pollution exposure.

Work-related exposure	Total<br>N = 151	< Secondary Education<br>N = 40	≥ Secondary Education<br>N = 111	p-value
Vehicle exhaust				0.5
not at all to rarely	15 (9.9%)	6 (15%)	9 (8.1%)	
some days	33 (22%)	8 (20%)	25 (23%)	
most days to daily	103 (68%)	26 (65%)	77 (69%)	
Cigarette smoke				0.8
not at all to rarely	66 (44%)	17 (43%)	49 (44%)	
some days	29 (19%)	9 (23%)	20 (18%)	
most days to daily	56 (37%)	14 (35%)	42 (38%)	
Marijuana smoke				0.4
not at all to rarely	70 (46%)	18 (45%)	52 (47%)	
some days	31 (21%)	11 (28%)	20 (18%)	
most days to daily	50 (33%)	11 (28%)	39 (35%)	
Unpaved/dusty roads				0.5
not at all to rarely	59 (39%)	13 (33%)	46 (41%)	
some days	43 (28%)	11 (28%)	32 (29%)	
most days to daily	49 (32%)	16 (40%)	33 (30%)	
Dumpsite				0.6
not at all to rarely	82 (54%)	21 (53%)	61 (55%)	
some days	23 (15%)	8 (20%)	15 (14%)	
most days to daily	46 (30%)	11 (28%)	35 (32%)	
Welding				0.2
not at all to rarely	86 (57%)	26 (65%)	60 (54%)	
some days	19 (13%)	2 (5.0%)	17 (15%)	
most days to daily	46 (30%)	12 (30%)	34 (31%)	
Wood cooking				0.2
not at all to rarely	98 (65%)	22 (55%)	76 (68%)	
some days	12 (7.9%)	5 (13%)	7 (6.3%)	
most days to daily	41 (27%)	13 (33%)	28 (25%)	
Kerosene cooking				0.005
not at all to rarely	93 (62%)	18 (45%)	75 (68%)	
some days	19 (13%)	4 (10%)	15 (14%)	
most days to daily	39 (26%)	18 (45%)	21 (19%)	
Rubbish burning				0.01
not at all to rarely	53 (35%)	11 (28%)	42 (38%)	
some days	61 (40%)	24 (60%)	37 (33%)	
most days to daily	37 (25%)	5 (13%)	32 (29%)	
Construction Dust				0.7
not at all to rarely	109 (72%)	27 (68%)	82 (74%)	
some days	14 (9.3%)	4 (10%)	10 (9.0%)	
most days to daily	28 (19%)	9 (23%)	19 (17%)	
Charcoal cooking				0.13
not at all to rarely	108 (72%)	24 (60%)	84 (76%)	
some days	19 (13%)	6 (15%)	13 (12%)	
most days to daily	24 (16%)	10 (25%)	14 (13%)	
Painting				0.5
not at all to rarely	110 (73%)	31 (78%)	79 (71%)	
some days	25 (17%)	7 (18%)	18 (16%)	
most days to daily	16 (11%)	2 (5.0%)	14 (13%)	
Factory/industry smoke				0.12
not at all to rarely	137 (91%)	35 (88%)	102 (92%)	
some days	7 (4.6%)	1 (2.5%)	6 (5.4%)	
most days to daily	7 (4.6%)	4 (10%)	3 (2.7%)	
Factory/industry waste				0.9
not at all to rarely	140 (93%)	37 (93%)	103 (93%)	
some days	6 (4.0%)	2 (5.0%)	4 (3.6%)	
most days to daily	5 (3.3%)	1 (2.5%)	4 (3.6%)	
Charcoal/brickmaking smoke				>0.9
not at all to rarely	151 (100%)	40 (100%)	111 (100%)	
some days	0 (0%)	0 (0%)	0 (0%)	
most days to daily	0 (0%)	0 (0%)	0 (0%)	

Pesticide/fertilizer				0.2
not at all to rarely	148 (98%)	38 (95%)	110 (99%)	
some days	3 (2.0%)	2 (5.0%)	1 (0.9%)	
most days to daily	0 (0%)	0 (0%)	0 (0%)	
