# Supplementary material for: Educational attainment and self-reported environmental exposures of pregnant women living in Nairobi, Kenya
Source: PLOS Glob Public Health. 2025 Nov 18;5(11):e0005453. doi: 10.1371/journal.pgph.0005453 (PMC12626295; doi:10.1371/journal.pgph.0005453)
Supplement: S2 File — Self-reported household fuels and sources of household air pollution exposure. S2 Table. Self-reported sources of ambient (outdoor) air pollution exposure. S3 Table. Self-reported sources of work-related air pollution exposure. (PDF) [file pgph.0005453.s004.pdf]

Participant ID Number: __ __ __ __ **Date: __ __ / __ __ __ /20 __ __**

Visit code: __ __ __ __

Site of Interview:

- Dandora 1
- Dandora 2
- Other, specify ________________

Screening ID Number: __ __ __ __

SOCIO-DEMOGRAPHICS

1. Age of mother__ __ yrs Date of birth__ __/ __ __ __ / __ __ __ __
   DD/MM/YYYY
2. Marital Status (tick one)
   - Married
   - Divorced/Separated
   - Widowed
   - Never married/ Single
   - Other Specify_____________________
3. Who is your main financial support person? (tick one)
   - Husband
   - Parent
   - Boyfriend
   - Other relative
   - None
   - Other Specify____________
4. What is your highest level of education? (tick one)
   - College/ University
   - Vocational
   - Secondary (Form_______)
   - Primary (Std _____)
   - Pre primary
5. What are the total numbers of years in school? ___________ yrs
6. What is your employment status? (tick one)
   - Monthly Salary
   - Daily Wage
   - Small Business
   - Homemaker (housewife)
   - Other specify____________-
   - None
7. Can you tell us your monthly rent? __ __ __ __ __Kes
   - This is private Information
   - Zero (own house)

HEALTH STATUS (RECORDED IN MCH BOOKLET OR CLIENT'S REPORT)

8. LMP: __ __/ __ __ __ / __ __ __ __ DD/MMM/YYYY
   - o Mother's self-report
   - o MCH booklet
   - o Unknown
9. EDD: __ __/ __ __ __ / __ __ __ __DD/MMM/YYYY
   - o Clinician estimate
   - o MCH booklet
   - o Unknown

10. Para__________+________ Gravida ______

11. What was your height prior to this pregnancy? __ __ __ . __ (cm)
    - o I don't know

12. What was your weight prior to this pregnancy? __ __ __. __(kg)
    - o I don't know

13. Have you lost any pregnancy? (select appropriately)
    - o Never Lost a pregnancy
    - o Miscarriage. (<28 weeks) Number____________
    - o Still Birth (>=28 weeks) Number _________________

14. Have you experienced any childhood deaths of your children?
    - o No
    - o Yes (No. of children______)
    - o N/A (no children)

15. Do you regularly consume stones or soil? (Tick all that apply)

    a. During this pregnancy
    Y/N Not consumed
    Y/N Stones
    Y/N Soil

    b. Before this pregnancy
    Y/N Not consumed
    Y/N Stones
    Y/N Soil

16. Did you have any challenges in the previous pregnancies? (indicate yes/no for each)
    Y/N Hyperemesis Gravidarum (Excessive vomiting with weight loss)
    Y/N Preeclampsia hypertension
    Y/N Eclampsia
    Y/N Premature rupture of membranes
    Y/N Cervical incompetence
    Y/N Gestational diabetes
    Y/N Urinary tract infection
    Y/N Ante-partum hemorrhage
    Y/N Post-partum hemorrhage
    Y/N Chorioamnionitis
    Y/N Severe abdominal cramps
    Y/N Depression
    Y/N Anxiety
    Y/N Other, specify____________
    Y/N None
    Y/N N/A (first pregnancy)

17. Problems in this current pregnancy?(indicate with a yes/no for each)

Y/N Hyperemesis gravidarum (excessive vomiting with weight loss)
Y/N Preeclampsia hypertension
Y/N Premature rupture of membranes
Y/N Cervical Incompetence
Y/N Gestational diabetes
Y/N Urinary tract infection
Y/N Ante-partum hemorrhage
Y/N Chorioamnionitis
Y/N Severe abdominal cramps
Y/N Depression
Y/N Anxiety
Y/N Other specify
Y/N None

DESCRIBING YOUR HOME

In this section we would like to ask you some questions about your house.

18. For how long have you stayed in your current house? ______ years _____ months
19. How many children in total reside/ stay with you? ___________________ (answer for all)

**Age group (yrs)**	**Number of children in age group specified**	**Age group (yrs)**	**Number of children in age group specified**
<1		6 to 9	
1 to 3		10 to 14	
4 to 5		15 to 17	

20. How many people in total (including participant) reside in your house? __________people
21. In total your house has how many:

    Rooms________ Windows (that open) __________ External doors ____________

22. Please describe the Kitchen/ room that is used for cooking in your home. Respond to all with a yes or no and state the number where applicable

		YES or NO	Number
a)	External doors		
b)	Internal doors		
c)	Windows that open		
d)	Chimney		
e)	Vents in the wall		
f)	Gap between the top of the wall and the roof that allows movement of air		
g)	Defects/ holes on the wall		
h)	Other Specify____________		

23. What is the household's main drinking and cooking water source? (tick any that apply)

Y/N Piped water in the house
Y/N Borehole
Y/N River water
Y/N Piped water outside house (shared)
Y/N Rain water
Y/N Water vendor
Y/N Other, specify ______________

24. Where does the household store their drinking and cooking water mainly? (tick any that apply)
    - Y/N Plastic container
    - Y/N Clay pot
    - Y/N Painted metal container
    - Y/N Non-painted metal container
    - Y/N Other, specify_____________

25. What type of toilet do you use?
    - o Pit latrine
    - o Flush toilet
    - o Other, specify ___________________

26. Provide details about the house structure (Indicate yes/no for each)

A. Roof		B. Walls		C. Floor		D. Floor covering	
	a) Wood		a) Wood		a) Wood		a) Vinyl sheet
	b) Mabati (silver)		b) Metal/iron sheets		b) Metal/iron sheets		b) Paint
	c) Treated/ corrugated iron		c) Red brick		c) Concrete		c) Red floor polish
	d) Tiles		d) Stone		d) Stone		d) Rug/carpet
	e) Concrete		e) Polythene		e) Red brick		e) Wood vanish
	f) Polythene		f) Mud/dung		f) PVC tiles		f) Other, sp_____
	e) Thatched/grass		g) Fabric		g) Ceramic tiles		g) None
	f) Other, sp______		h) Other, sp______		h) Soil/mud/dung		
					i) Other, sp_____		

27. Are the floors painted?
    - o Yes
    - o No

If yes, is the paint chipping/ breaking?
- o Yes
- o No

28. Are the walls painted?
    - o Yes
    - o No

If yes, is the paint chipping/ breaking?
- o Yes
- o No

29. Does your home have access to electricity?
    - o Yes
    - o No

30. Do you have the following in your home? (tick all that apply)

**Features of the Home**	Yes	No
a) Pets (cats, dogs, birds)		
b) Visible mold in home		
c) Mold odor in home (dampness)		
d) Cockroaches, rat problems		

PARTICIPANT'S ENVIRONMENTAL EXPOSURES

31. a) Indoor household exposures to pollutants from combustion/burning (tick all that apply)

**Cooking Fuels**		**Daily**	**Most days**	**Some days**	**Rarely**	**Not at all**
a	Wood/kuni					
b	Kerosene/parrafin					
c	Charcoal/makaa					
d	Slow burning charcoals/brickettes					
e	Koko fuel					
f	LPG gas					
g	Electricity					
h	Other, specify_____________					
**Other Sources of Household Pollution**						
a	Burning incense					
b	Insecticide sprays					
c	Kerosene lamp					
d	Burning mosquito repellant					
e	Cigarette smoke in the house					
f	Marijuana smoke in the house					
g	Candles (for lighting)					
h	Rubbish burning					
i	Other Specify__________					

b) How many people smoke cigarettes or marijuana in your home? _________________

32. a) How often do you cook just outside your house? (tick one)

- o Daily
- o Most days
- o Some days
- o Rarely
- o Not at all

b) What do you cook with when you cook just outside your house?

Y/N Wood/kuni
Y/N Kerosene/paraffin
Y/N Charcoal/makaa
Y/N Slow burning charcoal
Y/N Other, specify___________
Y/N N/A (do not cook outside the house)

33. Do you live near the following? (Indicate a yes/no for each)

Y/N Factory/industry
Y/N Garage activity
Y/N Y/N Battery recycling operators
Y/N Polluted river
Y/N Sewerage
Y/N E-waste recycling operators
Y/N Dumpsite
Y/N None of these
Y/N Other environmental pollutant source, please specify______________

34. Outdoor household (around the residence/home) exposures to pollutants from combustion/burning (answer for all)

**Air pollutant exposures close to the home (e.g. within 20 metres)**		**Daily**	**Most days**	**Some days**	**Rarely**	**Not at all**
a	Our own outdoor cooking smoke					
b	Neighbours cooking smoke					
**Pollutants from beyond the home (e.g. <1 kilometre)**						
c	Cooking smoke					
d	Vehicle smoke					
e	Unpaved roads					
f	Industry/ factory smoke					
g	Industry factory waste					
h	Dumpsite					
i	Rubbish burning					
j	Construction dust					
k	Smoke from making charcoal/ bricks					
l	Pesticides/ fertilizer spray					
m	Welding shop activity					
n	Painting work					
o	Other, specify (e.g. burning tires) ___________________					

35. a) Your own exposures from work (answer for all)

**Exposures**		**Daily**	**Most days**	**Some days**	**Rarely**	**Not at all**
a	Kerosene cooking					
b	Charcoal cooking					
c	Wood cooking					
d	Vehicle smoke					
e	Unpaved roads					
f	Factory/industry smoke					
g	Factory/industry waste					
h	Dumpsite					
i	Rubbish burning					
j	Construction dust					
k	Painting work					
l	Smoke from making charcoal/ bricks					
m	Pesticides/ fertilizer spray					
n	Welding shop activity					
o	Cigarette/marijuana smoke					
p	Other, specify __________________					

b) Apart from you, does anyone in your household work in any of these sectors? (respond with yes/no for each)

Y/N Battery recycling
Y/N Garage repair work (auto repair)
Y/N Electronic recycling
Y/N Dumpsite work
Y/N Welding
Y/N Painting job
Y/N Construction
Y/N Pesticides
Y/N Other, specify__________

36.Do you use any of these products?(respond with a yes/no for each)

Y/N Kohl
Y/N Skin lightening products
Y/N Other, specify

37.Do you apply kohl to your children?

- Yes
- No
